# Classifying Medulloblastoma Subgroups Based on Small, Clinically Achievable Gene Sets

**DOI:** 10.3389/fonc.2021.637482

**Published:** 2021-06-10

**Authors:** Sivan Gershanov, Shreyas Madiwale, Galina Feinberg-Gorenshtein, Igor Vainer, Tamar Nehushtan, Shalom Michowiz, Nitza Goldenberg-Cohen, Yehudit Birger, Helen Toledano, Mali Salmon-Divon

**Affiliations:** ^1^ Department of Molecular Biology, Ariel University, Ariel, Israel; ^2^ Hemato-Oncology Laboratory, Division of Pediatric Hematology Oncology, Schneider Children’s Medical Center of Israel, Petach Tikva, Israel; ^3^ Sackler Faculty of Medicine, Tel-Aviv University, Tel-Aviv, Israel; ^4^ Department of Pediatric Neurosurgery, Schneider Children’s Medical Center of Israel, Petach-Tikva, Israel; ^5^ Department of Ophthalmology, Bnai Zion Medical Center, Haifa, Israel; ^6^ The Krieger Eye Research Laboratory, Felsenstein Medical Research Center, Rabin Medical Center, Petach-Tikva, Israel; ^7^ The Ruth and Bruce Rappaport Faculty of Medicine, Technion, Haifa, Israel; ^8^ Department of Pediatric Oncology, Schneider Children’s Medical Center of Israel, Petach-Tikva, Israel; ^9^ Adelson School of Medicine, Ariel University, Ariel, Israel

**Keywords:** medulloblastoma, subgroup classification, biomarkers, machine learning, gene expression

## Abstract

As treatment protocols for medulloblastoma (MB) are becoming subgroup-specific, means for reliably distinguishing between its subgroups are a timely need. Currently available methods include immunohistochemical stains, which are subjective and often inconclusive, and molecular techniques—e.g., NanoString, microarrays, or DNA methylation assays—which are time-consuming, expensive and not widely available. Quantitative PCR (qPCR) provides a good alternative for these methods, but the current NanoString panel which includes 22 genes is impractical for qPCR. Here, we applied machine-learning–based classifiers to extract reliable, concise gene sets for distinguishing between the four MB subgroups, and we compared the accuracy of these gene sets to that of the known NanoString 22-gene set. We validated our results using an independent microarray-based dataset of 92 samples of all four subgroups. In addition, we performed a qPCR validation on a cohort of 18 patients diagnosed with SHH, Group 3 and Group 4 MB. We found that the 22-gene set can be reduced to only six genes (*IMPG2*, *NPR3*, *KHDRBS2*, *RBM24*, *WIF1*, and *EMX2*) without compromising accuracy. The identified gene set is sufficiently small to make a qPCR-based MB subgroup classification easily accessible to clinicians, even in developing, poorly equipped countries.

## Introduction

Medulloblastoma (MB)—the most common malignant brain tumor in children—demonstrates extremely high biological and clinical heterogeneity (1). Accordingly, it is divided into four subgroups, each representing distinct clinical, biological, and genetic profiles and involves a distinct activation pathway (2–7): WNT (or Group 1) involves Wingless pathway signaling (3); SHH (or Group 2) involves sonic hedgehog pathway signaling (4); Group C (or Group 3) involves photoreceptor and GABAergic pathway signaling; and Group D (or Group 4) involves neuronal and glutamatergic signaling (6). Importantly, although the histological presentation of the different subgroups is often similar, their response to treatment and the clinical outcomes are subgroup-specific (8); therefore, the World Health Organization has recently recommended that molecular markers be integrated as part of MB tumor diagnostic criteria (9). This recommendation is currently limited to distinguishing between the WNT and SHH subgroups, but means for distinguishing between Group 3 and Group 4 are already clinically required.

Transcriptomic analyses have shown promising potential for distinguishing between the four MB subgroups. Most notably, Northcott et al. (10) employed the NanoString technology that is based on a direct molecular barcoding of target molecules, followed by digital detection of their expression, to identify a set of 22 genes that can distinguish between the four MB subgroups (11); this set is currently used in many clinical laboratories worldwide. However, NanoString has two important limitations vis-à-vis its clinical use for MB subgroup classification: first, it is expensive and currently unavailable in most medical institutes, especially in developing countries; and second, it is not sufficiently reliable and shows relatively high rates of MB misdiagnosis and subgroup misclassification, especially between groups C and D (12). DNA methylation is more reliable in MB subgroup classification (13), but it is even more costly than NanoString and is unavailable in most medical institutes. Thus, there is a need to develop a reliable—yet simple and cost-effective—means of MB subgroup classification, which could be utilized through readily available technologies, such as qPCR. Indeed, Kunder et al. (14) used a quantitative PCR (qPCR) analysis, based on 21 biomarkers (including 12 protein-coding genes and nine microRNA expression profiles), but this number of genes is still high, hence impractical for qPCR test in the clinic.

To meet this need, this study aimed to identify sets of genes that comprise the minimal number of genes required for reliably differentiating between all four MB subgroups. To achieve this goal, we fed published data from microarray studies of MB, which comprehensively characterized the expression pattern of thousands of genes simultaneously, as input for machine-learning-based classifiers for cancer classification (15–17). Such classifiers were previously applied to discriminate anaplastic from non-anaplastic MB image regions (18) and to predict subtypes of the four MB subgroups (19), but, to the best of our knowledge, they have not been used to extract sets of potential biomarkers from microarray data. Indeed, this approach has enabled us to identify both protein-coding genes and non-coding RNAs as potential biomarkers for MB subgroup classification. These biomarkers could reliably be used in MB-related diagnosis, prognosis, and clinical decision-making, and they could later be used to identify potential drug targets.

## Methods

### Public Datasets

To identify minimal gene sets for MB subgroup classification, we used the dataset GSE85217 (19) to train and test the algorithms, and the datasets GSE37418 (20) and GSE41842 (21) for validation. All datasets are publicly available, quality-controlled, mRNA expression matrixes that were generated using Affymetrix microarrays. The datasets were downloaded from the gene expression omnibus (GEO) (22) database, which contains data on subjects diagnosed with any of the four MB subgroups. Specifically, the GSE85217 dataset comprises 763 samples (70 WNT samples, 223 SHH samples, 144 Group 3 samples, and 326 Group 4 samples), which were molecularly classified by inferring the expression levels of 22 MB signature genes, using the NanoString technology. The GSE37418 dataset comprises 73 samples (14 WNT samples, 13 SHH samples, 18 Group 3 samples, and 47 Group 4 samples), which were segregated into four MB subgroups using mRNA expression profiling and immunohistochemistry. The GSE41842 dataset comprises 19 samples (six WNT samples, three SHH samples, two Group 3 samples, and eight Group 4 samples), which were molecularly classified using unsupervised hierarchical clustering with the 1000 most differentially expressed genes. All samples included in these datasets were collected from fresh frozen tissue samples. Demographic and clinical data available for the above datasets is provided in **Supplementary File 1** - **Public Datasets**.

### Public Dataset Normalization

For the datasets GSE85217 and GSE41842, we downloaded the robust multi-array average normalized matrixes. For the GSE37418 dataset, we normalized the gene expression data by using the MAS 5.0 algorithm; therefore, we downloaded the raw CEL files and performed a robust multi-array average normalization by using the affy R package (23).

### Microarray Gene Annotation

To identify and match gene symbols to the probe ID of molecules in the two Affymetrix microarray datasets mentioned above, we used the biomaRt R package (24).

### Machine Learning Algorithms for Classification

We used the Waikato environment for knowledge analysis (WEKA) workbench software (25)—a Java-based machine learning algorithm collection—for all classification analyses. We initially employed four well-known algorithms: C4.5 Decision Tree (DT) (algorithm J48) (26); Decision Rules (RIPPER Rule Induction algorithm JRip) (27); Random Forest (28); and Support Vector Machines (SVM) using Sequential Minimal Optimization (SMO) (29–31). We chose the default parameters for all algorithms and used a 10-fold cross-validation to prevent overfitting. A detailed description of the methodology is provided in the **Supplementary Information** section.

In addition to the four well-established algorithms mentioned above, we designed and developed a novel algorithm that we termed SVM Attribute Ranking and Combinations (SARC). The main steps of the algorithm included: 1. building six pairwise models for the four MB subgroups, using the SVM classification model with a linear kernel; 2. for each binary classifier, ranking the attributes according to their squared weight; 3. for each subgroup, performing an aggregation of attribute ranks by summarizing each attribute rank to produce final ranks, leading to a list of top attributes; 4. using a combination of 0–12 top attributes (**Supplementary Table S1**) of each subgroup as the de-facto feature-selection method for the final classifier; and 5. producing an SVM classifier based on the 134 combinations, eventually choosing the smallest, best-performing combinations for each accuracy level. When using the NanoString 22-gene set to build the classification model, we used combinations of all 22 attributes. A more detailed description is provided in the Supplementary Information section (**Supplementary Tables S2**–**S5** and **Supplementary Figure S1**). We used the top nine reduced gene sets output by the SARC classifier (**Supplementary Table S3**) as input for the independent public dataset validation.

### Visualization

We generated clustering plots by using t-SNE, a non-linear dimensionality-reduction algorithm, with the Rtsne (32, 33) R package, version 0.15. Each plot was made with 1,000 iterations and the perplexity set to 30.

### Patient Cohort and Tumor Collection for Validation

An independent cohort of pediatric and young adult patients diagnosed with MB was collected at the Pediatric Hematology & Oncology Department at the Schneider Children’s Medical Center, Israel, and from the Pathology Department at the Rabin Medical Center, Israel. Since 2013, the standard of care has been to assign MB subgroup by using the NanoString nCounter Technology (NanoString Technologies, Seattle, WA), as described previously (10). We selected only the patients with MBs whose tumor subgroup had been classified by NanoString for clinical purposes and who had remaining RNA for real time PCR validation. Group-A MB (WNT) samples were not available to us, hence only SHH, Group 3 and Group 4 were included in qPCR analysis. The RNA was obtained from primary tumors for the initial clinical standard of care test at the time of diagnosis before any treatment; we did not extract any new RNA for this study. Altogether, the cohort used for validation comprised 18 children and young adults (8 males, 10 females; mean age at diagnosis: 6.53 ± 4.5 years), who were classified by NanoString as either SHH, Group 3, Group 4, or non-WNT/SHH (i.e., either Group 3 or Group 4) MBs (n = 5, 3, 8, and 2 respectively; **Supplementary Table S6**). Of the 18 patients, 11 were diagnosed with a localized disease and six were diagnosed with a metastatic disease (four M1 and two M2); data were unavailable for one patient (SHH4). All patients were treated with chemotherapy, eight patients underwent autologous bone marrow transplantation, and 14 patients received radiation therapy in addition to chemotherapy. Four patients did not receive radiation therapy due to their young age (<3 y). Disease recurrence was recorded in three patients. Four patients died altogether, including one who died from disease progression and three who died from other causes: patient SHH5 died as a result of secondary AML, patient C2 died of secondary diffuse intrinsic pontine glioma (DIPG) despite not receiving radiation, and patient D7 died from post-operative complications prior to therapy. All tissue samples, were from freshly frozen (FF) tissues. The study design adhered to the tenets of the Declaration of Helsinki and was approved by the local IRB and the National Review Board of the Israel Ministry of Health.

### Reverse-Transcription (RT) and qPCR

The cDNA synthesis was performed using the cDNA Reverse Transcription Kit (ABI High Capacity cDNA reverse-transcription kit, Cat No. 4368813) and was followed by a quantitative expression analysis using the SYBR Green qPCR Kit (PowerUP SYBR green master mix ABI, Cat No. A25776) according to the manufacturer’s instructions. The expression levels of each gene were normalized to those of *GAPDH*. Data and melting curves were analyzed by using the QuantStudio3 real-time instrument (Applied Biosystems, Waltham, Massachusetts(and associated software. Primer sequences are provided in **Supplementary Table S7**.

### qPCR Expression Level Analysis

The expression level of each protein-coding gene was normalized to that of GAPDH, as determined by the delta cycle threshold (dCt) method. Since we did not have a control (non-MB) cerebellum sample, we used dCt for unsupervised hierarchical clustering, generated using the pvclust (34) R package, version 2.0-0. Euclidean was used as the distance measure and ward.D2 was used as the linkage method. For each cluster in the dendrogram, p-values were calculated by multiscale bootstrap resampling (nboot = 1000).

## Results

### Applying Machine-Learning Algorithms for MB Subgroup Classification

To detect the minimal set of genes that accurately distinguishes between MB subgroups, we employed four well-known machine-learning algorithms, including Decision Tree, Decision Rules, Random Forest, and Support Vector Machines (SVM-SMO). The different algorithms were run in two modes. In the first, all 21,641 attributes (defined as Probe ID, **Supplementary File 2**) were used as input to the algorithm; in the second, the algorithms were fed with the known NanoString 22-gene set. The attributes selected by each algorithm for classification in either mode, as well as the classification accuracy, are indicated in **Table 1**. All four algorithms were highly accurate, as compared with the known 22-gene set of the NanoString panel. The Decision Tree and Decision Rules models resulted in a reduced gene sets (9 and 10 genes, respectively) with a similar or a slightly higher accuracy than that of the 22-gene signature set, while Random Forest and SVM-SMO used all input attributes and demonstrated the highest accuracy (**Table 1**).

**Table 1:** The accuracies of the sets of attributes selected for classification by each algorithm, based on the GSE85217 dataset (n = 763 MB samples).

Algorithm	Input^1^	Accuracy(%)	Attributes required for classification (output)^2^	Number of attributes required for classification
Decision tree^3^	All attributes	95.5	*OTX2, TMEM51, AIF1L, RASSF4, DYNC1I1, TRAK2, RPL3, C1orf112, RABGAP1*	9
	22 genes	94.5	*ATOH1, WIF1, RBM24, PDLIM3, NRL, TNC, GABRA5, KHDRBS2, SFRP1, IMPG2*	10
Decision rules^3^	All attributes	94.2	*PDLIM4, NPR3, PDE10A, PDK2, RALGPS2, SHD, BSG, ARNTL2, USP2, FBXL21*	10
	22 genes	94	*GAD1, PDLIM3, WIF1, EYA1, NPR3, EYS, RBM24, GABRA5, EOMES, EMX2, KCNA1, ATOH1, IMPG2*	13
Random forest	All attributes	97.8	* All attributes*	21,641
	22 genes	97.1	* All attributes*	22
SVM-SMO	All attributes	98.4	* All attributes*	21,641
	22 genes	97.8	* All attributes*	22

^1^Attribute sets that were used as inputs for the algorithm.

^2^Attributes chosen by each algorithm for classification.

^3^Detailed results obtained from these algorithms can be found in **Supplementary Figure S1** and **Supplementary Table S5**.

### The SVM Attribute Ranking and Combinations (SARC) Classifier Displays the Highest Accuracy

Despite the high accuracy of the Random Forest and SVM-SMO algorithms, they did not enable us to derive a gene-set output because they are non-interpretative regarding the attributes being used during the classification process. Therefore, we developed a novel algorithm—the SVM Attribute Ranking and Combinations (SARC)—in an attempt to obtain accuracy levels that are comparable to or higher than those of the Random Forest and SVM-SMO algorithms, while allowing a tailored feature selection.

**Figure 1:**
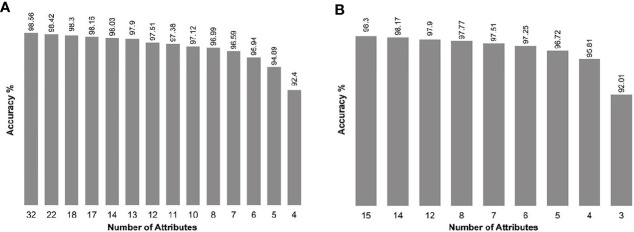
Accuracy of the smallest best-performing gene sets output by the SARC classifier, applied on the GSE85217 dataset (n = 763 samples), **(A)** when introducing all 21,641 attributes as input, and **(B)** when introducing the Nanostring 22-gene set as input.

When we introduced all genes as input, the SARC classifier provided a list of gene sets (between four and 32 biomarkers in each set; **Figure 1A** and **Supplementary Table S2**), of which the top 14 sets demonstrated accuracy levels between 92.4% and 98.56%. In most sets, the lowest number of genes necessary for classification was in the WNT and SHH group, while the highest number necessary was in Group 4. When we introduced the NanoString 22-gene set as input, the SARC classifier provided nine gene sets (**Figure 1B** and **Supplementary Table S3**) that comprised between three and 15 biomarkers and demonstrated an accuracy between 92.01% for the smallest set (three genes) and 98.3% for the largest set (15 genes). **Table 2** indicates the gene sets that demonstrated the highest accuracy levels; these include a set of 32 genes obtained when all genes were introduced to the SARC classifier as input, and a set of 15 genes obtained when the NanoString 22-gene set was introduced as input. Indeed, the SARC algorithm demonstrated the highest accuracy of all five tested algorithms.

**Table 2:** The accuracies of the top set of attributes selected for classification by the SARC algorithm for each input, based on the GSE85217 dataset (n = 763 MB samples).

Input^1^	Accuracy (%)	Attributes required for classification (output)^2^	Number of attributes required for classification
All attributes	98.6	*AL513318.2, NPR3, LMX1A, BARHL1, SIX6, GRM8, NID2, CA4, ZIC2, RBM24, ZIC5, DDX31, SNCAIP, NEUROG1, ATOH1, KCNA5, PEX5L, GLRA1, NDP, ZFHX4, RPGRIP1, PAX3, WIF1, TMEM51, ADGRL3, DLX3, TMEM51-AS1, TMEM132C, PGM5, PDE11A, NKD1, FZD10*	32
22 genes	98.3	*KHDRBS2, RBM24, EMX2, PDLIM3, NPR3, UNC5D, IMPG2, TNC, GABRA5, GAD1, OAS1, ATOH1, EYA1, EOMES, SFRP1*	15

^1^Attribute that were used as inputs for the algorithm.

^2^Attributes chosen by the algorithm for classification.

### The SARC Classifier Reduces the Number of Biomarkers Required for Accurate Classification to Only Six Genes – Validation in an Independent Dataset

The best-performing sets used by the SARC algorithm for classification comprised either 32 or 15 attributes. This number of biomarkers is too large to be practically used for qPCR in the clinic. The performances of the various reduced sets of genes (**Supplementary Tables S2**, **S3**) suggested that the number of biomarkers can be reduced to only six genes (*IMPG2*, *NPR3*, *KHDRBS2*, *RBM24*, *WIF1*, and *EMX2*) without compromising accuracy (**Supplementary Table S3** and **Supplementary Figure S2**). To validate this assumption, we tested the classification accuracy of these nine reduced sets (listed in **Supplementary Table S3**) in two independent public datasets, GSE37418 (20) and GSE41842 (21), which, together, contain 92 samples (73 and 19 samples, respectively) of all four MB subgroups. The classification accuracy of the six-gene set was 93.48%, which is higher than the accuracy observed when all 22 NanoString genes were introduced to the model (**Table 3**) accuracy, sensitivity, and specificity is specified in **Supplementary File 3** – **Confusion Matrix**.

**Table 3:** Classification accuracy of the reduced genes sets (12 genes or fewer), as compared with the full, 22-gene NanoString set, used on the independent validation datasets GSE37418 and GSE41842 (n = 92 MB samples altogether).

Number of attributes	Accuracy (%)	Input set for validation^1^
22	91.30	*EYS, TNC, IMPG2, OAS1, EYA1, SFRP1, KCNA1, RBM24, KHDRBS2, NPR3, GAD1, NRL, PDLIM3, DKK2, WIF1, UNC5D, EOMES, HHIP, EMX2, ATOH1, MAB21L2, GABRA5*
12	96.74	*IMPG2, NPR3, EMX2, RBM24, SFRP1, NRL, TNC, PDLIM3, KHDRBS2, UNC5D, ATOH1, WIF1*
8	90.22	*IMPG2, KHDRBS2, RBM24, EMX2, PDLIM3, NPR3, UNC5D, WIF1*
7	93.48	*IMPG2, KHDRBS2, RBM24, EMX2, PDLIM3, NPR3, WIF1*
6	93.48	*IMPG2, NPR3, KHDRBS2, RBM24, WIF1, EMX2*
5	82.61	*IMPG2, NPR3, KHDRBS2, RBM24, WIF1*
4	81.52	*IMPG2, KHDRBS2, RBM24, WIF1*

^1^Attribute sets that were used as input for the validation based on the SARC classifier output, chosen from the GSE85217 dataset (**Supplementary Table S3**).

**Figure 2:**
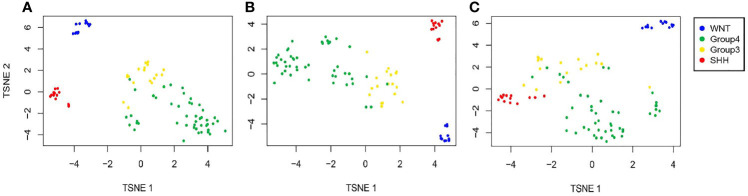
Validation of the predicted classification set outputs created by the SARC classifier. Expression t-SNE of the independent datasets GSE37418 and GSE41842 (n = 92) based on **(A)** a 22-gene NanoString panel set, **(B)** 12 genes out of the 22 Nanostring panel, and **(C)** six genes out of the 22 Nanostring panel.

Next, we created t-SNE plots (**Figure 2**) to visualize the performance of the full NanoString and the reduced gene sets on the validation dataset (n = 92 samples). Both gene sets performed well in separating the MB groups, with a slightly better separation observed by the 12-gene set, whose performance was similar to that of the full 22-gene set. Not surprisingly, the WNT and SHH groups are presented as clearly separated clusters, while the separation between Group 3 and Group 4 is less pronounced.

### Classifying MB Subgroups in an Independent Clinical Cohort Based on the SARC Reduced Gene Set, Using qPCR

**Figure 3:**
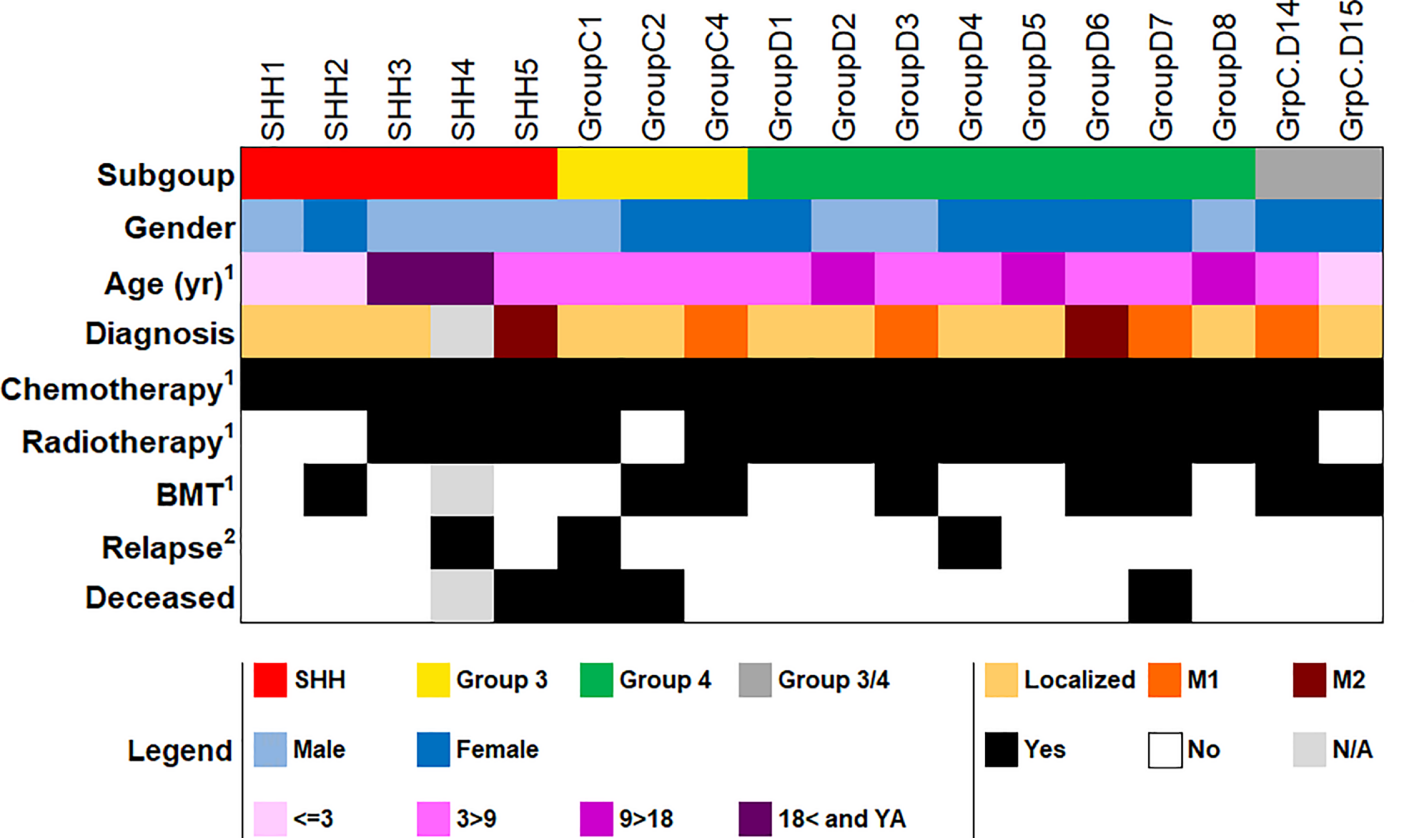
Demographic and clinical data of the patient cohort used for qPCR validation (n = 18). BMT, bone marrow transplantation; YA, young adult; N/A, not available. ^1^At first diagnosis. ^2^As of the completion of this study. More detailed information in **Supplementary Table S6**.

As a proof-of-concept that the suggested gene sets can be used to classify MB subgroups in patients by using gene expression levels generated by qPCR, we validated our results on an independent cohort of 18 patients, whose MB subgroup was previously classified by NanoString. The cohort included five patients with SHH MB, three patients with Group 3 MB, eight patients with Group 4 MB, and two patients who were classified as non-WNT/SHH MB, i.e., with either Group 3 or Group 4 MB (**Figure 3** and **Supplementary Table S6**). At the time of completion of this study, we did not have samples from patients with a WNT MB; hence, this subgroup was not included in the validation.

The unsupervised hierarchical clustering was performed using the expression levels (namely, dCt) of the reduced six-gene set (*IMPG2*, *NPR3*, *KHDRBS2*, *RBM24*, *WIF1*, and *EMX2*).

**Figure 4:**
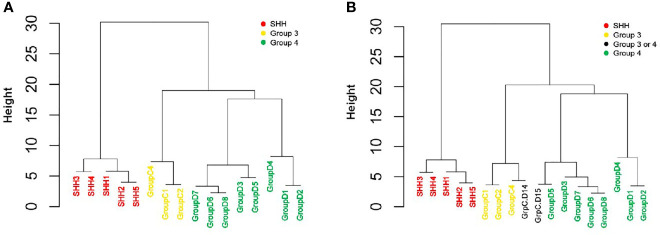
qPCR-based classification of an independent cohort, using reduced six-gene setout of the 22-gene NanoString set (*IMPG2*, *NPR3*, *KHDRBS2*, *RBM24*, *WIF1*, and *EMX2*). An unsupervised hierarchical clustering of gene expression levels was generated by using qPCR (dCt) values. **(A)** A cohort of 16 patients who were classified by NanoString as having either SHH, Group 3, or Group 4 MBs (n = 5, 3, and 8, respectively; see **Figure 3** and **Supplementary Table S6**). **(B)** The same cohort, but with the addition of two patients who were classified as having a non-WNT/SHH MB. The Height (y axis) is a measure of closeness of either individual data points or clusters.

The reduced gene set performed well in classifying the patients to their diagnosed MB subgroups (**Figure 4A** and **Supplementary Figure S3A**). Adding the two patients whose subgroup was undefined resulted in the clustering of patient GrpC.D14 with patients from Group 3, and of patient GrpC.D15 with patients from Group 4 (**Figure 4B** and **Supplementary Figure S3B**). Hence, our data demonstrate the potential of using this small set of genes for an easy and accessible qPCR-based MB subgroup classification.

## Discussion

Using feature selection and machine learning classification, we were able to identify potential gene sets with fewer attributes and a more accurate subgroup classification of MB tumors, as compared with the NanoString 22-gene set currently used in several clinical institutions. More specifically, our SARC algorithm was able to reduce the 22-gene set to only six genes that reliably differentiated between the four MB subgroups. The reduced gene set includes *WIF1* and *EMX2* which are known activated Wingless pathway signaling in WNT subgroup. Widely accepted biomarkers *IMPG2*, and *NPR3* identity Group 3, as well as *KHDRBS2*, and *RBM24* recognise Group 4 MB tumors (10). Notably, none of these genes are classical biomarkers of SHH subgroup, and probably the combination of these genes’ expression contributes to accurate SHH group classification.

All genes in this set are known and have commercially available primers, which should enable most clinical laboratories to accurately classify MB subgroups at a reasonable price and within a reasonable timeframe, to the benefit of both patients and clinicians alike.

The tumor subgroups in the GSE85217 dataset that we used to construct the model were originally determined according to the expression levels of the 22 genes by the NanoString technology. Hence, it was not surprising that the accuracy levels of all tested algorithms were very high when they were based on this 22-gene set. However, in the independent validation datasets, the subgroups were classified by using a different approach: in the GSE41842, the subgroups were classified according to unsupervised hierarchical clustering using the 1000 most differentially expressed genes, while in GSE37418, the subgroups were classified using the mRNA expression of 2,750 probes with the highest median absolute difference (MAD) score and with immunohistochemistry to provide an additional assessment for WNT and SHH subgroups (20, 21). Therefore, the high accuracy obtained at the validation step demonstrates the promising potential of using fewer biomarkers, such as 12 or six genes having higher accuracy (96.74% and 93.48% respectively) than the 22-gene set (91.3%). This potential was further demonstrated by the qPCR-based classification that we obtained by using the reduced six-gene set in the cohort of 22 pediatric patients. We included in this qPCR validation two patients whose subgroup was defined as “non-SHH/WNT”, one clustered with patients from Group 3, and one clustered with patients from Group 4. Methylation may help to determine the subgroup of these patients, to check if the reduced gene set model classified them correctly. Unfortunately, methylation was unavailable at the Schneider Children’s Medical Center as it is in most clinical centers. Future studies on larger cohorts are required to test the effectiveness of the reduced six-gene set in decreasing MB misclassification, in general, and in accurately distinguishing between Group 3 and Group 4 MBs, in particular.

Our study has several limitations; first, due to a lack of WNT samples, we were unable to add this subgroup to the qPCR validation step. Nevertheless WNT subgroup is easily identifiable by other currently available methods, e.g. using a combination of immunohistochemistry for nucleopositive beta-catenin, and FISH for monosomy of chromosome 6 (35). Future studies should use qPCR to test the reduced gene set of all MB subgroups. Second, since both our modeling and validation steps were performed on primary tumors, we cannot comment on the performance of the reduce set on metastasis, relapse, or progression disease samples. Third, our models do not distinguish between the different subtypes of each subgroup; instead, the algorithm was trained to classify the different subgroups regardless of their molecular states, especially since the current clinical recommendations focus only on the main subgroups and do not consider the different subtypes. Future studies should take intertumoral heterogeneity within MB subgroups into consideration. Finally, the current study focused on the minimal set of genes required for MB subgroup classification, but implementation in a clinical setting requires that the suggested gene set is adapted to an individual patient setting. Such a setting should include a cut-off of the detection of expression level for each gene, a definition of the reference that should be used, a statement of the type of normalization that should be employed, etc.

## Conclusions

Since personalized treatment in oncology assumes that each tumor harbors a unique variation of the human genome and should be treated accordingly, it is crucial to correctly classify the molecular subgroup of the tumor. Indeed, as treatment (e.g., radiation and chemotherapy) protocols are becoming subgroup-specific and usually commence within 28 days of operation, our machine-learning approach, which yielded concise and reliable gene sets, provides a significant clinical advantage over available MB subgroup classification methods.

## Data Availability Statement

The original contributions presented in the study are included in the article/**Supplementary Material**. Further inquiries can be directed to the corresponding author.

## Ethics Statement

The study design adhered to the tenets of the Declaration of Helsinki and was approved by the local IRB and the National Review Board of the Israel Ministry of Health. Written informed consent from the participants’ legal guardian/next of kin was not required to participate in this study in accordance with the national legislation and the institutional requirements.

## Author Contributions

Conceptualization: SG and MS-D. Formal analysis: SG, IV, and TN. Investigation: SG, HT, and MS-D. Methodology: SG, IV, and MS-D. Resources: GF-G, SMi, NG-C, and HT. Supervision: YB, HT, and MS-D. Validation: SMa, GF-G, and YB. Visualization: SG. Writing – original draft: SG, HT, and MS-D. Writing – review and editing: SG, SM, GF-G, IV, TN, NG-C, YB, HT, and MS-D. All authors contributed to the article and approved the submitted version.

## Funding

This study was funded by the Levi Eshkol Fund, Ministry of Science, Technology & Space, Israel, grant number 3-12624, which provided SG’s scholarship.

## Conflict of Interest

The authors declare that the research was conducted in the absence of any commercial or financial relationships that could be construed as a potential conflict of interest.
