# Supplementary material for: Classifying Medulloblastoma Subgroups Based on Small, Clinically Achievable Gene Sets
Source: Front Oncol. 2021 Jun 10;11:637482. doi: 10.3389/fonc.2021.637482 (PMC8223061; doi:10.3389/fonc.2021.637482)
Supplement: Supplementary file 1 [file DataSheet_1.docx]

Supplementary Material

**1. Supplementary Materials and Methods**

For all classification analyses, we used the Waikato Environment for Knowledge Analysis (WEKA) workbench software (1): a collection of Java-based machine learning algorithms.

For the C4.5 Decision Tree (DT) algorithm J48 (2), the default parameters were chosen to build small and compact pruned trees. The validation was accomplished using 10-fold cross-validation to prevent overfitting. Notably, the final tree only uses some of the genes that we have provided to the algorithm, while, during the building process, it most probably used more than the final set. Nevertheless, only the final set of genes is required for the final classification model.

For the RIPPER Rule Induction algorithm JRip (3), the default parameters were chosen to build the pruned Decision Rules model. The validation was accomplished using 10-fold cross-validation. The final model is applied by reading rules from top to bottom. The classification of an unclassified example is the first matching rule from the list of the rules produced by the algorithm, adhering to the example in question. The last rule provides a class for "all else" – if none of the previous rules match, we fall back to the default decision on this class.

For the Random Forest algorithm (4), the default parameters were chosen to build the model. The classification model was built using 10-fold cross-validation.

For the Support Vector Machines (SVM) using Sequential Minimal Optimization (SMO) (5–7), the default parameters were chosen to build the model. The classification model was built using 10-fold cross-validation. The SMO implementation is very fast and robust, using the linear kernel trick. While the algorithm works on binary-class problems only, pairwise classification is used on multi-class problems.

1.1. SVM attribute ranking and combinations (SARC)

When an SVM classification model is trained using a linear kernel, each attribute is assigned a weight, used in conjunction with all other attributes to produce support vectors to comprise the ultimate model. The weight of an individual attribute in such a classification model indicates the importance of its contribution to the performance of the classifier algorithm. The weights assigned to an attribute can be either positive or negative, but their absolute (squared) values can help in assessing the contribution of each attribute to the classification task (8,9). Previous studies have shown the potential of utilizing the weight interpretation of linear SVM for different objectives (10–12). Thus, in the method proposed here, we interpreted the squared values of assigned weights as indicators of the potential of each attribute to contribute to the successful discrimination between MB classes.

Using SVM classification model with a linear kernel, we built six pairwise models for four classes. For every binary classifier, we ranked the attributes according to their squared weight. For every class, an aggregation of attribute ranks was performed by summarizing the ranks of every attribute to produce final ranks, leading to a list of top attributes. Then, we used a combination of 0 to 12 top attributes of every class as the de-facto feature-selection method for the final classifier (Table S1). We then produced an SVM classifier based on the 134 combinations, eventually choosing the smallest, best-performing combinations for each accuracy level. When using the 22-gene signature set to build the classification model, we used combinations of all 22 attributes.

1.1.1. Detailed methodology

1. For every cartesian combination {*Group3_TOP_, Group4_TOP_, SHH_TOP_, WNT_TOP_*}, each assigned a value in the range of {*0, …, 12*}:
   1. Apply a standard *10-fold cross-validation* framework, where the data are randomly divided into 10 equally sized parts (folds)
   2. For each fold:
      1. Define the nine remaining folds as the train dataset *F_TRAIN_* and hold-out the current fold as the test dataset *F_TEST_*
      2. On the train dataset *F_TRAIN_*:
         1. Produce a *Linear kernel SVM* classification model *M*, comprised of six pairwise *linear kernel binary SVM* models, *M_BINARY_C_i_C_j_*, where *i, j* in {*1, …, 4*}
         2. For every class *C_i_*, produce a list of ranked attributes using *M*:
            1. For every attribute *A_n_* of *C_i_*, *C_i_A_n_*:

Initialize SumOfRanks_C_i_A_n_ = 0

- - - - 1. For each of the three remaining classes, *C_j_*:

Choose the respective linear kernel binary SVM model M_BINARY_C_i_C_j_ between C_i_ and C_j_

Interpret the attribute weights given by M_BINARY_C_i_C_j_:

Order the attributes by their descending squared weight (top attributes having the highest squared weights)

Rank attributes by their order (e.g., the first attribute in the ordered list is assigned *rank=1*, the second is assigned *rank=2*, etc.)

Assign: *AttributeRank_A_n_ =* attribute’s rank

Ties are assigned the same rank

For every attribute *C_i_A_n_*:

SumOfRanks_C_i_A_n_ = SumOfRanks_C_i_A_n_ + AttributeRank_A_n_

- - - - 1. Order the attributes by ascending sums of ranks (i.e., the attributes on the top of the list have the smallest *SumOfRanks* values)

Ties are assigned the same rank

- - - 1. Choose {*Group3_TOP_, Group4_TOP_, SHH_TOP_, WNT_TOP_*} attributes per each respective class, as per the evaluated combination, adhering to the list of ranked attributes produced in ‎(2)
      2. Produce a *linear kernel SVM* classification model *M_TOP_* using only the retained top attributes as per the evaluated combination
    1. Apply model *M_TOP_* on the hold-out test set *F_TEST_*
    2. Evaluate the model by its accuracy (percentage of correctly classified instances)
  1. Aggregate and average the accuracies between all 10 folds to produce the final accuracy score for the evaluated combination

1. Per each accuracy level (e.g., accuracy result of 98.5%, 97.9%, etc.), starting from maximal achieved accuracy down to the lower bound of 90%:
   1. Return the smallest combination of attributes yielding the level’s accuracy
2. The final result is a table listing the smallest combination of top-performing attributes, yielding the highest-accuracy classification results.

The 12 genes detected for each class are presented in Table S1. The top 12 genes were found to be unique for each class. Out of the 48 detected genes, six genes (*ATOH1, GABRA5, MAB21L2, NPR3, RBM24,* and *WIF1*) are also included in the 22-gene signature set. In addition, we found the long non-coding RNA (lncRNA) *AL513318.2,* also known as the uncharacterized *LOC440173* gene, which was ranked as the top gene in Group 3. Once all 13^4^ combinations (0–12 genes in each of the four subgroups) were tested, we listed the smallest, best-performing combinations for each accuracy level (Table S2). For the highest accuracy level (98.6%), the number of attributes was 32 (WNT = 11, SHH = 8, Group 3 = 2, Group 4 = 11), which was higher than expected for the WNT and SHH attributes. However, for all other accuracy levels, the highest number of attributes was in Group 4 and the lowest was in the WNT and SHH groups (Table S2).

When using the 22-gene signature set as an input to the SARC algorithm, the highest observed accuracy was 98.3% and the number of attributes decreased from 22 to 15 (Table S3). For comparison, for the same accuracy levels, we obtained only 13 attributes (WNT = 1, SHH = 2, Group 3 = 3, Group 4 = 7) when all genes were used as input to SARC classifier (Table S3). The 32 genes that yielded the highest accuracy were subjected to a gene-set enrichment analysis (geneanalytics.genecards.org) (13). Of these 32 genes, 13 genes (Npr3, Kcna5, Neurog1, Zic2, Six6, Pex5l, Zic5, Fzd10, PAX3, Zfhx4, Atoh1, Wif1, and Barhl1) are expressed in the medulla oblongata and 11 genes (KCNA5, Neurog1, Zic2, Pex5l, RPGRIP1, Zic5, Fzd10, PAX3, ZFHX4, Atoh1, Barhl1) are expressed in the cerebellum. The top gene ontology processes of these genes were visual perception, multicellular organism development, nervous system development, and neuron differentiation (Table S4).

The first ranked attribute in the WNT sub-group in the SARC model is the gene *PAX3*, a crucial transcription factor for melanocyte development (14), which was shown to be highly expressed in MB (15). Despite evidence of direct regulation of *PAX3* by the WNT signaling pathway (16,17), *PAX3* has not been used, to date, as a biomarker for MB WNT tumors. The second ranked attribute in WNT sub-group is the gene *WIF1*, which is a known biomarker for the MB WNT tumors, included in the 22-gene signature set and in a qPCR analysis set (18).

In the SHH sub-group, the top two ranked attributes found by the SARC model, *NEUROG1* and *ATOH1*, are known to be related to MB. *NEUROG1* is a transcription factor that is expressed in MB lacking *ATOH1* expression (19)*. ATOH1* is included in the 22-gene signature set. *ATOH1* is overexpressed in MB driven by aberrant SHH pathway (20–23), and a mouse MB model study revealed that *ATOH1* promotes metastasis in MB SHH tumors (24). Figure 2b demonstrates that *NEUROG1* has a low expression predominantly in the SHH sub-group, whereas *ATOH1* has a high expression level.

For Group 3, the first ranked attribute in the SARC model is a long non-coding RNA, *AL513318.2*, also known as the uncharacterized *LOC440173* gene. In recent years, it has become clear that the non-protein-coding portion of the genome has an important functional role in normal development and disease (reviewed in (25)), as well as in regulating gene expression (reviewed in (26)). Types of non-coding RNAs (ncRNAs) include long RNA (lncRNAs) and short RNAs, such as microRNAs (miRNAs), small interfering RNAs (siRNAs), and Piwi-interacting RNAs (piRNAs). To date, studies in MB have used specific candidate miRNA approaches and microarray expression profiling techniques, which implicated a series of candidate biomarkers (27–33). Some of these biomarkers have been found to have a role in the MB subgroups biology, such as the lncRNA that promotes the growth and metastasis of medulloblastoma (34–36). To the best of our knowledge, no relationship has been previously found between the lncRNA *LOC440173* and MB, either in general or with respect to a specific sub-group. The well-known Group 3 biomarker *NPR3* is the second ranked attribute for this group.

In the SARC model of Group 4, the fourth ranked attribute is the *GRM8* gene, whose expression level is used as a marker to distinguish Group 3 from Group 4 MB tumors (18). The first ranked attribute is the LIM homeobox transcription factor 1 (*LMX1A*), whose enhancer activity and expression was high in Group 4 tumors (37).

*1.2. Decision Trees (DT)*

When all genes were introduced to the DT algorithm as input, we yielded a classification model of nine genes (*OTX2, TMEM51, AIF1L, RASSF4, DYNC1I1, TRAK2, RPL3, C1orf112,* and *RABGAP1*)—none of which is included in the 22-gene signature set—with an accuracy of 95.5% (Figure S1a). When using the 22-gene signature set to build the tree model, 10 genes (*ATOH1, WIF1, RBM24, PDLIM3, NRL, TNC, GABRA5, KHDRBS2, SFRP1,* and *IMPG2*) were required for the classification, yielding an accuracy of 94.5% (Figure S1b).

*1.3. Decision Rules*

When all genes were introduced to the DR algorithm as input, we yielded a classification set of seven rules (Table S5) and a classification model of 10 genes (*PDLIM4, NPR3, PDE10A, PDK2, RALGPS2, SHD, BSG, ARNTL2, USP2,* and *FBXL21*)—which are different from those of the DT classification model—with an accuracy of 94.2% (Table S5). When using the 22-gene signature set to build a collection of eight rules using 13 genes (*GAD1, PDLIM3, WIF1, EYA1, NPR3, EYS, RBM24, GABRA5, EOMES, EMX2, KCNA1, ATOH1,* and *IMPG2*), a similar accuracy of 94.0% was obtained.

Both the DT and the Decision Rules models resulted in a reduced gene sets (9 and 10 genes, respectively) with a similar or a slightly higher accuracy than that of the 22-gene signature set. The first node in the tree produced by the DT algorithm is the *OTX2* gene. *OTX2* is overexpressed in the WNT, Group 3, and Group 4 tumors, and is under-expressed in the SHH subgroup. Thus, *OTX2* has been used as a biomarker to differentiate the SHH from other MB subgroups (18). Other genes present in the tree, such as *RPL3*, *AIF1L*, *DYNC1I1*, and *RABGAP1*, are indirectly related to MB and are related to *TP53*. For example, a decreased degradation of the p53 protein was observed in cell lines after a small interfering RNA-mediated knockdown of *RPL31* (38), and *RABGAP1* preferentially interacts with mutant *TP53 (p53R273H)* (39). Somatic *TP53* mutations in patients with MB have a poor long-term survival (40), in particular among SHH MB (41). One gene out of the set of seven rules resulting from the Decision Rules model is *NPR3*, which appears in the 22-gene signature set and in a qPCR analysis set (18). Prior to the use of transcriptome analysis for classifying MB tumors, positive *NPR3* immunohistochemistry staining of formalin-fixed tumors was used to diagnose Group 3 MB (42).

# Supplementary Figures and Tables

## Supplementary Tables

# Table S1. Top 12 ranking attributes out of all gene sets produced by SVM classifier for every class that were used in the SARC classifier.

| **Rank** | **WNT** | **SHH** | **Group 3** | **Group 4** |
| --- | --- | --- | --- | --- |
| 1 | PAX3 | NEUROG1 | AL513318.2 (LOC440173) | LMX1A |
| 2 | WIF1 | ATOH1 | NPR3 | BARHL1 |
| 3 | TMEM51 | KCNA5 | PPP2R2B | SIX6 |
| 4 | ADGRL3 | PEX5L | ROBO3 | GRM8 |
| 5 | DLX3 | GLRA1 | RNY3P10 | NID2 |
| 6 | TMEM51-AS1 | NDP | GABRA5 | CA4 |
| 7 | TMEM132C | ZFHX4 | PAPPA2 | ZIC2 |
| 8 | PGM5 | RPGRIP1 | PAX6 | RBM24 |
| 9 | PDE11A | RSPO3 | THNSL2 | ZIC5 |
| 10 | NKD1 | CPNE4 | RASGRF2 | DDX31 |
| 11 | FZD10 | STX3 | ARHGEF28 | SNCAIP |
| 12 | NOL4 | OTX2 | MAB21L2 | SIX1 |

# Table S2. The smallest gene sets for every accuracy level of each group in the SARC classifier when introducing all 21,641 attributes as input.

| **Accuracy^1^** | **Total # of att.** | **WNT** | **SHH** | **Group 3** | **Group 4** | **Attributes^2^** |
| --- | --- | --- | --- | --- | --- | --- |
| 98.56 | 32 | 11 | 8 | 2 | 11 | PAX3, WIF1, TMEM51, ADGRL3, DLX3, TMEM51-AS1, TMEM132C, PGM5, PDE11A, NKD1, FZD10, NEUROG1, ATOH1, KCNA5, PEX5L, GLRA1, NDP, ZFHX4, RPGRIP1, AL513318.2 (LOC440173), NPR3, LMX1A, BARHL1, SIX6, GRM8, NID2, CA4, ZIC2, RBM24, ZIC5, DDX31, SNCAIP |
| 98.42 | 22 | 1 | 8 | 5 | 8 | PAX3, NEUROG1, ATOH1, KCNA5, PEX5L, GLRA1, NDP, ZFHX4, RPGRIP1, AL513318.2 (LOC440173), NPR3, PPP2R2B, ROBO3, RNY3P10, LMX1A, BARHL1, SIX6, GRM8, NID2, CA4, ZIC2, RBM24 |
| 98.30 | 18 | 1 | 3 | 4 | 10 | PAX3, NEUROG1, ATOH1, KCNA5, AL513318.2 (LOC440173), NPR3, PPP2R2B, ROBO3, LMX1A, BARHL1, SIX6, GRM8, NID2, CA4, ZIC2, RBM24, ZIC5, DDX31 |
| 98.16 | 17 | 1 | 1 | 5 | 10 | PAX3 , NEUROG1, AL513318.2 (LOC440173), NPR3, PPP2R2B, ROBO3, RNY3P10, LMX1A, BARHL1, SIX6, GRM8, NID2, CA4, ZIC2, RBM24, ZIC5, DDX31 |
| 98.03 | 14 | 1 | 1 | 5 | 7 | PAX3, NEUROG1, AL513318.2 (LOC440173), NPR3, PPP2R2B, ROBO3, RNY3P10, LMX1A, BARHL1, SIX6, GRM8, NID2, CA4, ZIC2 |
| 97.90 | 13 | 1 | 2 | 3 | 7 | PAX3, NEUROG1, ATOH1, AL513318.2 (LOC440173), NPR3, PPP2R2B, LMX1A, BARHL1, SIX6, GRM8, NID2, CA4, ZIC2 |
| 97.51 | 12 | 1 | 2 | 2 | 7 | PAX3, NEUROG1, ATOH1, AL513318.2 (LOC440173), NPR3, LMX1A, BARHL1, SIX6, GRM8, NID2, CA4, ZIC2 |
| 97.38 | 11 | 1 | 1 | 2 | 7 | PAX3, NEUROG1, AL513318.2 (LOC440173), NPR3, LMX1A, BARHL1, SIX6, GRM8, NID2, CA4, ZIC2 |
| 97.12 | 10 | 1 | 1 | 4 | 4 | PAX3, NEUROG1, AL513318.2 (LOC440173), NPR3, PPP2R2B, ROBO3, LMX1A, BARHL1, SIX6, GRM8 |
| 96.99 | 8 | 1 | 1 | 4 | 2 | PAX3, NEUROG1, AL513318.2 (LOC440173), NPR3, PPP2R2B, ROBO3, LMX1A, BARHL1 |
| 96.59 | 7 | 1 | 1 | 3 | 2 | PAX3, NEUROG1, AL513318.2 (LOC440173), NPR3, PPP2R2B, LMX1A, BARHL1 |
| 95.94 | 6 | 1 | 1 | 2 | 2 | PAX3, NEUROG1, AL513318.2 (LOC440173), NPR3, LMX1A, BARHL1 |
| 94.89 | 5 | 1 | 1 | 2 | 1 | PAX3, NEUROG1, AL513318.2 (LOC440173), NPR3, LMX1A |
| 92.40 | 4 | 1 | 1 | 2 | 0 | PAX3, NEUROG1, AL513318.2 (LOC440173), NPR3 |

^1^Accuracy percentage, starting from maximum down to the lower bound of 90%

^2^The attributes are listed according to their ranking produced by SVM classifier that was used in the SARC classifier, from top to bottom

# Table S3. The smallest best-performing gene sets for every accuracy level of every class in the SARC classifier when introducing the Nanostring 22-gene set as input.

| **Accuracy^1^** | **Number of att.^2^** | **Attributes^3^** |
| --- | --- | --- |
| 98.30 | 15 | *KHDRBS2, RBM24, EMX2, PDLIM3, NPR3, UNC5D, IMPG2, TNC, GABRA5, GAD1, OAS1, ATOH1, EYA1, EOMES, SFRP1* |
| 98.17 | 14 | *KHDRBS2, RBM24, EMX2, PDLIM3, NPR3, UNC5D, IMPG2, TNC, GABRA5, GAD1, OAS1, ATOH1, EYA1, EOMES* |
| 97.90 | 12 | *IMPG2, NPR3, EMX2, RBM24, SFRP1, NRL, TNC, PDLIM3, KHDRBS2, UNC5D, ATOH1, WIF1* |
| 97.77 | 8 | *IMPG2, KHDRBS2, RBM24, EMX2, PDLIM3, NPR3, UNC5D, WIF1* |
| 97.51 | 7 | *IMPG2, KHDRBS2, RBM24, EMX2, PDLIM3, NPR3, WIF1* |
| 97.25 | 6 | *IMPG2, NPR3, KHDRBS2, RBM24, WIF1, EMX2* |
| 96.72 | 5 | *IMPG2, NPR3, KHDRBS2, RBM24, WIF1* |
| 95.81 | 4 | *IMPG2, KHDRBS2, RBM24, WIF1* |
| 92.01 | 3 | *IMPG2, KHDRBS2, RBM24,* |

^1^Accuracy percentage, starting from maximum down to the lower bound of 90%

^2^The smallest combination of attributes yielding the level’s accuracy

^3^The attributes are listed according to their ranking produced by SVM classifier that was used in the SARC classifier, from top to bottom

# Table S4. Function-based and Gene Ontology (GO)-biological process analysis of the 32 attributes performing the highest accuracy level in the SARC classifier

| **GO-biological process** | **Number of att.^1^** | **Attributes** |
| --- | --- | --- |
| Visual Perception | 6 | SIX6, NDP, GLRA1, GRM8, RPGRIP1, ZIC2 |
| Multicellular Organism Development | 10 | FZD10, SIX6, NEUROG1, WIF1, DLX3, ATOH1, ZIC2, ZIC5, PAX3, LMX1A |
| Nervous System Development | 7 | NDP, NEUROG1, ATOH1, BARHL1, ZIC2, ZIC5, PAX3 |
| Neuron Differentiation | 4 | FZD10, NEUROG1, ATOH1, LMX1A |

^1^ Number of attributes

**Table S5.** Decision Rules model results.

| **#** | **Role^1^** | **Class** | **Support^2^** |
| --- | --- | --- | --- |
| 1 | *PDLIM4* ≥ 7.55 | WNT | 72/2 |
| 2 | *NPR3* ≥ 5.56 and *PDE10A* ≤ 6.30 and *PDK2* ≤ 9.02 | Group 3 | 110/2 |
| 3 | *RALGPS2* ≥ 8.61 and *SHD* ≤ 6.35 | Group 3 | 27/1 |
| 4 | *BSG* ≥ 11.54 and *ARNTL2* ≤ 6.03 | Group 3 | 11/4 |
| 5 | *USP2* ≥ 10.30 | Group 3 | 2/0 |
| 6 | *FBXL21* ≤ 5.83 | SHH | 220/0 |
| 7 | All else | Group 4 | 321/0 |

^1^The model is applied by reading rules from top to bottom

^2^Support numbers represent the correct/incorrect classification of the model

**Table S6.** Demographic and clinical data of the patient cohort for the qPCR validation.

| **Pt. no.** | **Gen** | **Age (yr)^1^** | **Diagnosis** | **Loc/ Met** | **Relapse^2^** | **Deceased** | **Chemo- therapy^1^** | **Radio- therapy^1^** | **BMT^1^** | | **Tissue** |
| --- | --- | --- | --- | --- | --- | --- | --- | --- | --- | --- | --- |
| SHH1 | M | 1.25 | SHH | L | No | No | ACNS 1221 | No | No | FF | |
| SHH2 | F | 1 | SHH | L | No | No | COG 99703 | No | Yes | FF | |
| SHH3 | M | 20.3 | SHH | L | No | No | ACNS 0331 | Yes | No | FF | |
| SHH4 | M | YA | SHH | N/A | Yes | N/A | Yes | Yes | N/A | FF | |
| SHH5 | M | 4.5 | SHH | M2 | No | Yes | COG 99703 | Yes | No | FF | |
| GroupC1 | M | 7.5 | Group 3 | L | Yes | Yes | ACNS 0331 | Yes | No | FF | |
| GroupC2 | F | 3.2 | Group 3 | L | No | Yes | COG 99703 | No | Yes | FF | |
| GroupC4 | F | 4 | Group 3 | M1 | No | No | SJMB 03 | Yes | Yes | FF | |
| GroupD1 | F | 5.9 | Group 4 | L | No | No | ACNS 0331 | Yes | No | FF | |
| GroupD2 | M | 10 | Group 4 | L | No | No | ACNS 0331 | Yes | No | FF | |
| GroupD3 | M | 3.7 | Group 4 | M1 | No | No | SJMB 03 | Yes | Yes | FF | |
| GroupD4 | F | 7.9 | Group 4 | L | Yes | No | ACNS 0331 | Yes | No | FF | |
| GroupD5 | F | 10.1 | Group 4 | L | No | No | ACNS 0331 | Yes | No | FF | |
| GroupD6 | F | 7.3 | Group 4 | M2 | NO | No | SJMB 03 | Yes | Yes | FF | |
| GroupD7 | F | 7.1 | Group 4 | M1 | No | Yes | SJMB 03 | Yes | Yes | FF | |
| GroupD8 | M | 10.3 | Group 4 | L | No | No | ACNS 0331 | Yes | No | FF | |
| GrpC.D14 | F | 4 | Group 3 or 4 | M1 | No | No | SJMB 03 | Yes | Yes | FF | |
| GrpC.D15 | F | 3 | Group 3 or 4 | L | No | No | COG 99703 | No | Yes | FF | |

^1^ At first diagnosis.

^2^ As of completion of this study.

Gen, Gender; L, localized; Met, metastatic; BMT, bone marrow transplantation; YA, young adult; N/A, not available; FF, fresh frozen;

**Table S7**. primers sequence (hy-labs) for validation using quantitative PCR.

| Gene symbol | Forward sequence | Reverse sequence |
| --- | --- | --- |
| WIF1 | CCTGCCATGAACCCAACAAATGCC | GAGGCTGGCTTCGTACCTTTTATTGC |
| EMX2 | GCTCATCCACCGCTACCGATA | TTCTCAAAGGCGTGTTCCAGCC |
| NPR3 | CTAGGAGCTGGCTTGCTAAT | GGGTTCGCCTCTCAATGGTTAT |
| IMPG2 | TGTACTTGAATTTAGGTCCCCC | CCTCACCATTGAAGGTAACTGC |
| RBM24 | GAACCTGGCATACTTAGGAGCA | AGGTCTTTGTATAAGGGCTGGA |
| KHDRBS2 | CCCTCGGGGAAGCACTGTA | TGGGTCGCATAGCTGTTATCAT |

##
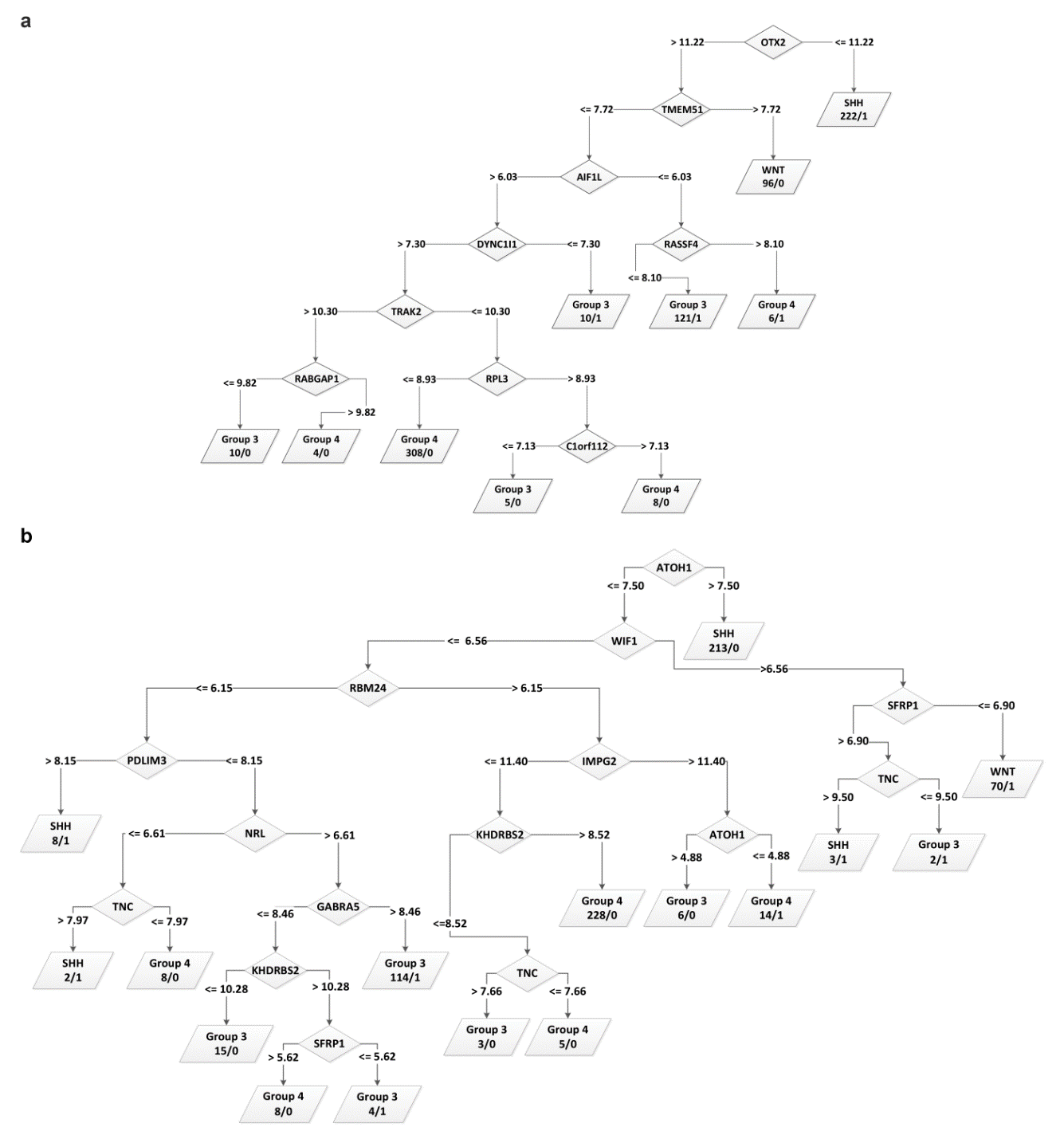
 Supplementary Figures

**Supplementary Figure S1.** Decision tree classification model. (a) All genes were provided to the algorithm, resulting in a classification model of nine genes. (b) A 22-gene signature set was used to build the model, resulting in a classification model of 10 genes. Support numbers under the class name represent the correct/incorrect classification rates of the model.


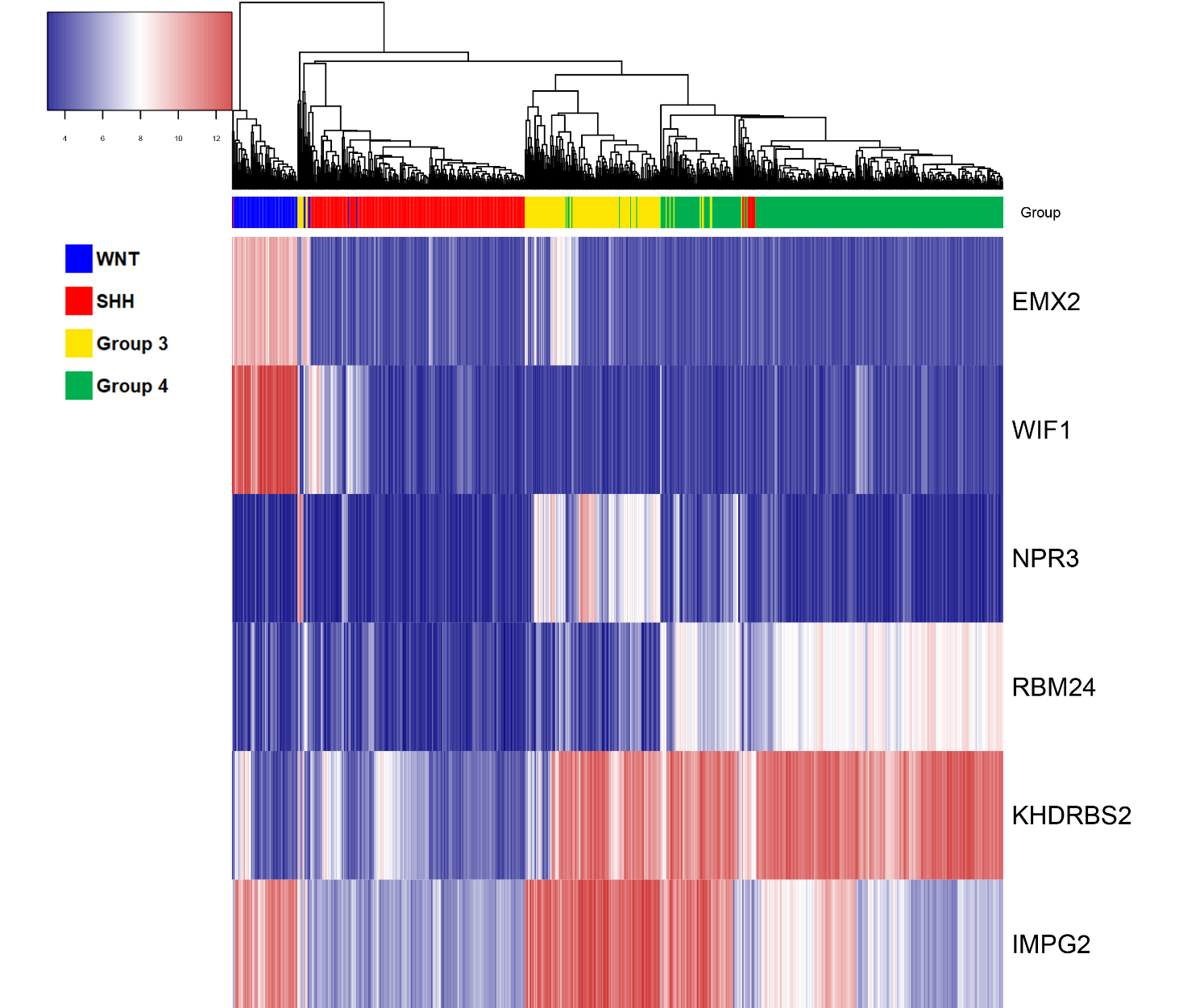


**Supplementary Figure S2.** Expression heat-maps applied on the GSE85217 dataset (n = 763) of the reduced six-gene set out of the NanoString 22-gene set.

The heatmap generated using ‘heatmap3’ R package, version 1.1.1, unsupervised hierarchical clustering (average linkage), with Euclidean as the distance method


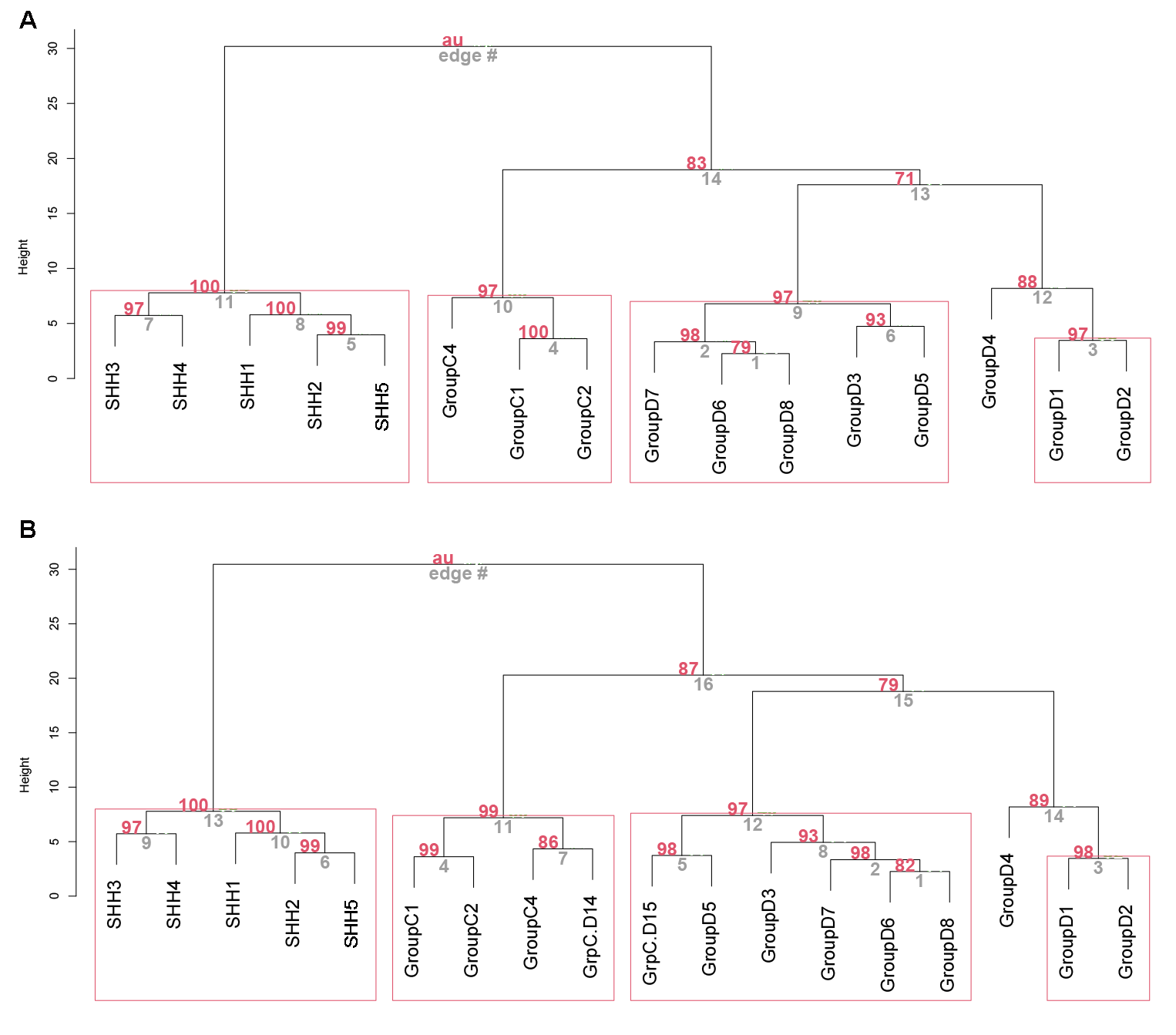


**Supplementary Figure S3.** Validation of the predicted classification gene set; qPCR-based classification of an independent cohort, using reduced six-gene setout of the 22-gene NanoString set (*IMPG2*, *NPR3*, *KHDRBS2*, *RBM24*, *WIF1*, and *EMX2*). Unsupervised hierarchical clustering of gene expression levels were generated by using qPCR (dCT) values. Red values on the edges are Approximately Unbiased (AU) p-values (%), and gray values indicate the edge number. Clusters with AU higher than 95%, which are strongly supported by data, are highlighted by red rectangles. (A) A cohort of 16 patients who were classified by NanoString as having either SHH, Group 3, or Group 4 MBs (n = 5, 3, and 8, respectively; see Figure 3 and Supplementary Table S6). (B) The same cohort, but with the addition of two patients who were classified as having a non-WNT/SHH MB.

**References**

1. Frank E, Hall MA, Witten IH. The WEKA Workbench. *Morgan Kaufmann, Fourth Ed* (2016)553–571. doi:10.1016/B978-0-12-804291-5.00024-6

2. Salzberg SL. C4.5: Programs for Machine Learning by J. Ross Quinlan. Morgan Kaufmann Publishers, Inc., 1993. *Mach Learn* (1994) **16**:235–240. doi:10.1007/BF00993309

3. Cohen WW. Fast effective rule induction. *Proc Twelfth Int Conf Mach Learn* (1995) **95**:115–123. doi:10.1.1.50.8204

4. Breiman L. Random forests. *Mach Learn* (2001) **45**:5–32. doi:10.1023/A:1010933404324

5. Platt JC. “Fast training of support vector machines using sequential minimal optimization,” in *Advances in Kernel Methods - Support Vector Learning*, 185–208. doi:10.1109/ISKE.2008.4731075

6. Keerthi SS, Shevade SK, Bhattacharyya C, Murthy KRK. Improvements to Platt’s SMO Algorithm for SVM Classifier Design. *Neural Comput* (2001) **13**:637–649. doi:10.1162/089976601300014493

7. Hastie T, Tibshirani R. Classification by pairwise coupling. *Ann Stat* (1998) **26**:451–471. doi:10.1214/aos/1028144844

8. Guyon I, Weston J, Barnhill S, Vapnik V. Gene selection for cancer classification using support vector machines. *Mach Learn* (2002) doi:10.1023/A:1012487302797

9. Guyon I, Elisseeff A. An Introduction to Variable and Feature Selection. *J Mach Learn Res* (2003) doi:10.1016/j.aca.2011.07.027

10. Samb ML, Camara F, Ndiaye S, Slimani Y, Esseghir MA. A Novel RFE-SVM-based Feature Selection Approach for Classification. *Int J Adv Sci Technol* (2012) **43**:27–36.

11. Chang Y-W, Lin C-J. Feature ranking using linear svm. *Causation Predict Challenge, Challenges Mach Learn* (2008)

12. Ding Y, Wilkins D. Improving the performance of SVM-RFE to select genes in microarray data. *BMC Bioinformatics* (2006) doi:10.1186/1471-2105-7-S2-S12

13. Ben-Ari Fuchs S, Lieder I, Stelzer G, Mazor Y, Buzhor E, Kaplan S, Bogoch Y, Plaschkes I, Shitrit A, Rappaport N, et al. GeneAnalytics: An Integrative Gene Set Analysis Tool for Next Generation Sequencing, RNAseq and Microarray Data. *Omi A J Integr Biol* (2016) **20**:139–151. doi:10.1089/omi.2015.0168

14. Zhang H, Chen H, Luo H, An J, Sun L, Mei L, He C, Jiang L, Jiang W, Xia K, et al. Functional analysis of Waardenburg syndrome-associated PAX3 and SOX10 mutations: Report of a dominant-negative SOX10 mutation in Waardenburg syndrome type II. *Hum Genet* (2012) doi:10.1007/s00439-011-1098-2

15. Gershon TR, Oppenheimer O, Chin SS, Gerald WL. Temporally regulated neural crest transcription factors distinguish neuroectodermal tumors of varying malignancy and differentiation. *Neoplasia* (2005) doi:10.1593/neo.04637

16. Degenhardt KR, Milewski RC, Padmanabhan A, Miller M, Singh MK, Lang D, Engleka KA, Wu M, Li J, Zhou D, et al. Distinct enhancers at the Pax3 locus can function redundantly to regulate neural tube and neural crest expressions. *Dev Biol* (2010) doi:10.1016/j.ydbio.2009.12.030

17. Boudjadi S, Chatterjee B, Sun W, Vemu P, Barr FG. The expression and function of PAX3 in development and disease. *Gene* (2018) **666**:145–157. doi:10.1016/J.GENE.2018.04.087

18. Kunder R, Jalali R, Sridhar E, Moiyadi A, Goel N, Goel A, Gupta T, Krishnatry R, Kannan S, Kurkure P, et al. Real-time PCR assay based on the differential expression of microRNAs and protein-coding genes for molecular classification of formalinfixed paraffin embedded medulloblastomas. *Neuro Oncol* (2013) **15**:1644–1651. doi:10.1093/neuonc/not123

19. Salsano E, Croci L, Maderna E, Lupo L, Pollo B, Giordana MT, Consalez GG, Finocchiaro G. Expression of the neurogenic basic helix-loop-helix transcription factor NEUROG1 identifies a subgroup of medulloblastomas not expressing ATOH1. *Neuro Oncol* (2007) doi:10.1215/15228517-2007-014

20. Ayrault O, Zhao H, Zindy F, Qu C, Sherr CJ, Roussel MF. Atoh1 inhibits neuronal differentiation and collaborates with Gli1 to generate medulloblastoma-initiating cells. *Cancer Res* (2010) doi:10.1158/0008-5472.CAN-09-3740

21. Dubuc AM, Northcott PA, Kenney AM, Taylor MD. Calculating a cure for cancer: Managing medulloblastoma MATH1-ematically. *Expert Rev Neurother* (2010) doi:10.1586/ern.10.126

22. Grimmer MR, Weiss WA. BMPs oppose Math1 in cerebellar development and in medulloblastoma. *Genes Dev* (2008) doi:10.1101/gad.1657808

23. Flora A, Klisch TJ, Schuster G, Zoghbi HY. Deletion of Atoh1 disrupts sonic hedgehog signaling in the developing cerebellum and prevents medulloblastoma. *Science (80- )* (2009) doi:10.1126/science.1181453

24. Grausam KB, Dooyema SDR, Bihannic L, Premathilake H, Morrissy AS, Forget A, Schaefer AM, Gundelach JH, Macura S, Maher DM, et al. ATOH1 promotes leptomeningeal dissemination and metastasis of sonic hedgehog subgroup medulloblastomas. *Cancer Res* (2017) doi:10.1158/0008-5472.CAN-16-1836

25. Esteller M. Non-coding RNAs in human disease. *Nat Rev Genet* (2011) **12**:861–874. doi:10.1038/nrg3074

26. Choudhuri S. Small noncoding RNAs: Biogenesis, function, and emerging significance in toxicology. *J Biochem Mol Toxicol* (2010) **24**:195–216. doi:10.1002/jbt.20325

27. Northcott PA, Fernandez-L A, Hagan JP, Ellison DW, Grajkowska W, Gillespie Y, Grundy R, Van Meter T, Rutka JT, Croce CM, et al. The miR-17/92 polycistron is up-regulated in sonic hedgehog-driven medulloblastomas and induced by N-myc in sonic hedgehog-treated cerebellar neural precursors. *Cancer Res* (2009) **69**:3249–3255. doi:10.1158/0008-5472.CAN-08-4710

28. Ferretti E, De Smaele E, Miele E, Laneve P, Po A, Pelloni M, Paganelli A, Di Marcotullio L, Caffarelli E, Screpanti I, et al. Concerted microRNA control of Hedgehog signalling in cerebellar neuronal progenitor and tumour cells. *EMBO J* (2008) **27**:2616–2627. doi:10.1038/emboj.2008.172

29. Garzia L, Andolfo I, Cusanelli E, Marino N, Petrosino G, De Martino D, Esposito V, Galeone A, Navas L, Esposito S, et al. MicroRNA-199b-5p impairs cancer stem cells through negative regulation of HES1 in medulloblastoma. *PLoS One* (2009) **4**: doi:10.1371/journal.pone.0004998

30. Uziel T, Karginov F V., Xie S, Parker JS, Wang Y-DY-D, Gajjar A, He L, Ellison D, Gilbertson RJ, Hannon G, et al. The miR-17~92 cluster collaborates with the Sonic Hedgehog pathway in medulloblastoma. *Proc Natl Acad Sci U S A* (2009) **106**:2812–7. doi:10.1073/pnas.0809579106

31. Cho Y-JJ, Tsherniak A, Tamayo P, Santagata S, Ligon A, Greulich H, Berhoukim R, Amani V, Goumnerova L, Eberhart CG, et al. Integrative genomic analysis of medulloblastoma identifies a molecular subgroup that drives poor clinical outcome. *J Clin Oncol* (2011) **29**:1424–1430. doi:10.1200/JCO.2010.28.5148

32. Weeraratne SD, Amani V, Neiss A, Teider N, Scott DK, Pomeroy SL, Cho YJ. miR-34a confers chemosensitivity through modulation of MAGE-A and p53 in medulloblastoma. *Neuro Oncol* (2011) **13**:165–175. doi:10.1093/neuonc/noq179

33. Bai AHC, Milde T, Remke M, Rolli CG, Hielscher T, Cho YJ, Kool M, Northcott PA, Jugold M, Bazhin A V., et al. MicroRNA-182 promot es leptomeningeal spread of non-sonic hedgehog-medulloblastoma. *Acta Neuropathol* (2012) **123**:529–538. doi:10.1007/s00401-011-0924-x

34. Gao R, Zhang R, Zhang C, Zhao L, Zhang Y. Long noncoding RNA CCAT1 promotes cell proliferation and metastasis in human medulloblastoma via MAPK pathway. *Tumori J* (2017)0–0. doi:10.5301/tj.5000662

35. Song H, Han L-M, Gao Q, Sun Y, Song H, Han L-M, Sun Y. Long non-coding RNA CRNDE promotes tumor growth in medulloblastoma.

36. Zhang H, Wang X, Chen X. Potential Role of Long Non-Coding RNA ANRIL in Pediatric Medulloblastoma Through Promotion on Proliferation and Migration by Targeting miR-323. *J Cell Biochem* (2017) **118**:4735–4744. doi:10.1002/jcb.26141

37. Lin CY, Erkek S, Tong Y, Yin L, Federation AJ, Zapatka M, Haldipur P, Kawauchi D, Risch T, Warnatz H-JJ, et al. Active medulloblastoma enhancers reveal subgroup-specific cellular origins. *Nature* (2016) **530**:57–62. doi:10.1038/nature16546

38. Maruyama Y, Miyazaki T, Ikeda K, Okumura T, Sato W, Horie-lnoue K, Okamoto K, Takeda S, Inoue S. Short hairpin RNA library-based functional screening identified ribosomal protein l31 that modulates prostate cancer cell growth via p53 pathway e108743. *PLoS One* (2014) **9**: doi:10.1371/journal.pone.0108743

39. Coffill CR, Muller PAJ, Oh HK, Neo SP, Hogue KA, Cheok CF, Vousden KH, Lane DP, Blackstock WP, Gunaratne J. Mutant p53 interactome identifies nardilysin as a p53R273H-specific binding partner that promotes invasion. *EMBO Rep* (2012) **13**:638–644. doi:10.1038/embor.2012.74

40. Tabori U, Baskin B, Shago M, Alon N, Taylor MD, Ray PN, Bouffet E, Malkin D, Hawkins C. Universal poor survival in children with medulloblastoma harboring somatic TP53 mutations. *J Clin Oncol* (2010) **28**:1345–1350. doi:10.1200/JCO.2009.23.5952

41. Zhukova N, Ramaswamy V, Remke M, Pfaff E, Shih DJH, Martin DC, Castelo-Branco P, Baskin B, Ray PN, Bouffet E, et al. Subgroup-specific prognostic implications of TP53 mutation in medulloblastoma. *J Clin Oncol* (2013) doi:10.1200/JCO.2012.48.5052

42. Northcott PA, Korshunov A, Witt H, Hielscher T, Eberhart CG, Mack S, Bouffet E, Clifford SC, Hawkins CE, French P, et al. Medulloblastoma comprises four distinct molecular variants. *J Clin Oncol* (2011) **29**:1408–1414. doi:10.1200/JCO.2009.27.4324
